# Inhibition of Human Cytochrome P450 Enzymes by Allergen Removed *Rhus verniciflua* Stoke Standardized Extract and Constituents

**DOI:** 10.1155/2014/150351

**Published:** 2014-06-30

**Authors:** Hyunsik Jung, Sanghun Lee

**Affiliations:** Department of Medical Consilience, Graduate School, Dankook University, 152, Jukjeon-ro, Suji-gu, Yongin-si, Gyeonggi-do 448-701, Republic of Korea

## Abstract

*Objective*. Potential interactions between herbal extracts and the cytochrome P450 (CYP) system lead to serious adverse events or decreased drug efficacy. *Rhus verniciflua* stoke (RVS) and its constituents have been reported to have various pharmacological properties. We evaluated the inhibitory potential of RVS and its constituents on the major CYP isoforms. *Methods*. The effects of allergen removed RVS (aRVS) standardized extract and major components, fustin and fisetin isolated from aRVS, were evaluated on CYP1A2, CYP2C9, CYP2C19, CYP2D6, and CYP3A4 isoenzyme activity by a luminescent CYP recombinant human enzyme assay. *Results*. The aRVS extract showed relative potent inhibitory effects on the CYP2C9 (IC_50_, <0.001 *μ*g/mL), CYP2C19 (IC_50_, 9.68 *μ*g/mL), and CYP1A2 (IC_50_, 10.0 *μ*g/mL). However, it showed weak inhibition on CYP3A4 and CYP2D6. Fustin showed moderate inhibitory effects on the CYP2C19 (IC_50_, 64.3 *μ*g/mL) and weak inhibition of the other CYP isoforms similar to aRVS. Fisetin showed potent inhibitory effects on CYP2C9, CYP2C19, and CYP1A2. Fisetin showed moderate inhibition of CYP2D6 and weak inhibition of CYP3A4. *Conclusions*. These results indicate that aRVS, a clinically available herbal medicine, could contribute to herb-drug interactions when orally coadministered with drugs metabolized by CYP2C9, CYP2C19, and CYP1A2.

## 1. Introduction

Nowadays, the paradigm of chemotherapies in cancer patients has changed with the development of chemotherapy such as capecitabine and targeted agents including tyrosine kinase inhibitors (TKI) and mammalian target of rapamycin (mTOR) inhibitors [1, 2]. These orally administered chemotherapeutic agents prescribed in the treatment of numerous cancers are metabolized by hepatic cytochrome P450 enzymes (CYP). Therefore, drug interaction in cancer patients must be noted because it can lead to overdosing or undertreatment resulting in severe clinical consequences compared to traditional time-limited cytotoxic chemotherapy [3, 4]. In addition, most herbal medicines which are orally administered could affect the activity of the intestinal affecting bioavailability of coadministered drugs [5, 6]. In addition, several herbs can give rise to the potential of harmful interactions with the targeted agents [7].

**Figure 1:**
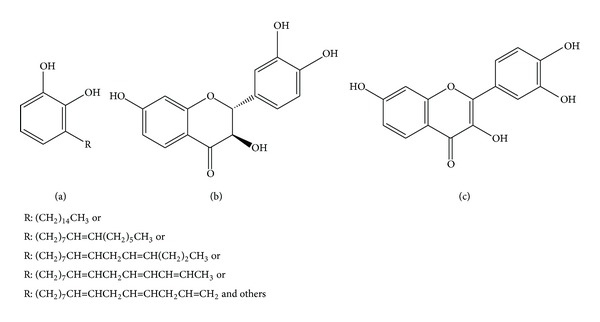
Structures of* Rhus verniciflua* stokes constituents. (a) Urushiol: the likelihood and severity of allergic reaction to urushiol are dependent on the degree of unsaturation of the alkyl chain. (b) Fustin: 2-(3,4-dihydroxyphenyl)-3,7-dihydroxy-2,3-dihydrochromen-4-one. (c) Fisetin, 2-(3,4-dihydroxyphenyl)-3,7-dihydroxychromen-4-one.

*Rhus verniciflua* stokes (RVS) of the Anacardiaceae family, commonly known as the lacquer tree, have been used in Korean medicine for centuries to treat diseases of the digestive system, including tumors [8]. Several preclinical studies of its flavonoids and other extracts have reported that it has antioxidant, anti-inflammatory, antiproliferative, and anti-cancer activities [9–18]. However, the clinical application of RVS has been restricted because urushiol (Figure 1(a)) can cause an allergic skin rash on contact, known as urushiol-induced contact dermatitis in sensitive individuals [19]. Therefore, urushiol, which is a mixture of several derivatives of catechol, should be removed from RVS for pharmaceutical use. Recently, several clinical reports of a standardized extract with the allergen removed RVS (aRVS) based on traditional method showed promising outcomes in advanced cancer patients [20–25]. Therefore, the potential effects of aRVS-drug interactions should be tested for the patients with concurrent chemotherapy.

In this study, aRVS standardized extract and some of its major and commercially available constituents, including fustin (>13.0% in aRVS) and fisetin (>2.0% in aRVS) (Figures 1(b) and 1(c)), were selected to study their inhibitory effects on the five major CYP isoforms (CYP1A2, CYP3A4, CYP2C9, CYP2C19, and CYP2D6) involved in the hepatic metabolism of most drugs.

## 2. Materials and Methods

### 2.1. Plant Materials

The standardized extract from RVS was prepared as follows. RVS stalk, which was 10 years old and grew in Wonju, Republic of Korea, was dried without exposure to direct sunlight and chopped up. The pieces were extracted two times with a 10-fold volume of water at 90°C to 95°C for 6 hours according to Korean patent no. 0504160. The extract was filtered with Whatman GF/B filter paper and concentrated under vacuum to remove water. The concentrate was lyophilized to a brownish powder. The extract yield from 100 g of chopped material was 3.3 g. A component analysis method using high performance liquid chromatography showed that the aRVS extract contained fustin, fisetin, sulfuretin, and butein, among others. The quality of the aRVS extract was tested and controlled according to the quality standards of the Korea Food & Drug Administration and our hospital's standards (fustin > 13.0%, fisetin > 2.0%, and urushiol not detected) [8]. The standard aRVS samples were comprised of 8 serial dilutions of aRVS, ranging between 0.001–10,000 *μ*g/mL dissolved in 50% methanol. Fustin (3,3′,4′,7-Tetrahydroxyflavanone, C_15_H_12_O_6_) is a flavonoid and constitutes 13–30% (w/w) of aRVS. The fustin extracted from aRVS by high-performance liquid chromatography mass spectroscopy had a purity of >95%. The standard samples were comprised of 8 serial dilutions of fustin, ranging between 0.001 and 10,000 *μ*g/mL dissolved in 10% methanol. The fisetin (3,3′,4′,7-Tetrahydroxyflavone, C_15_H_10_O_6_) is also a flavonoid and constitutes 2–5% (w/w) of aRVS. The fisetin was purchased from Sigma Chemical Co. (St. Louis, MO, USA) with a purity of >95%, because the fisetin extracted from aRVS was hard to ensure its purity. The standard samples were comprised of 8 serial dilutions of fisetin, ranging between 0.001 and 5,000 *μ*g/mL dissolved in 70% methanol.

### 2.2. Luminogenic P450 Enzymes Inhibition Assay

The assay was carried out using P450-Glo Screening system from Promega (Promega Inc., Madison Wis., USA). It contains recombinant human CYP1A2, CYP2C9, CYP2C19, CYP2D6, and CYP3A4 enzymes in the membranes produced by a baculovirus expression system, specific luminogenic cytochrome enzyme substrates (luciferin 6-methyl ether, luciferin 6-benzyl ether, 6-deoxyluciferin, ethylene glycol ester of 6-deoxyluciferin, and ethylene glycol ester of luciferin 6-methyl ether), negative control membranes, a nicotinamide adenine dinucleotide phosphate hydrogen (NADPH) regeneration system containing NADP+, glucose-6-phosphate, magnesium chloride (MgCl_2_) and glucose-6-phosphate dehydrogenase (functioning to initiate and sustain the CYP450 reaction by maintaining a non-limiting NADPH system), reaction buffer, luciferin detection reagent, and Luciferin-Free Water. The negative control membranes were devoid of CYP activity. The luminogenic inhibition assays were performed following the protocols from Promega Corp. (Technical Bulletin, P450-Glo Assays, Promega Corp., 2009) and previous studies [26, 27].

Samples of different concentrations of both the aRVS extract and the two compounds were prepared. Firstly, 12.5 *μ*L of the 4× aRVS extract or the test compounds (appropriate for each enzyme) was added to the “treated” wells. The 12.5 *μ*L of the luciferin-free water was added individually in both the “untreated” wells representing the values of total CYP activity and the “minus-P450 control” wells representing the values of the CYP-independent background luminescence. Secondly, 12.5 *μ*L of the 4× reaction mixture (containing human CYP membrane preparations, the appropriate luminogenic substrate, and potassium phosphate buffer) was added to the “treated” and the “untreated” wells. The 12.5 *μ*L of the 4× control reaction mixture (containing membrane preparations devoid of CYP enzymes, the appropriate luminogenic substrate, and potassium phosphate buffer) was added to the “minus-P450 control” wells. The plate was preincubated at room temperature (25°C) for 10 min, and then 25 *μ*L of the 2× NADPH regeneration system was added to initiate the reaction. After this was incubated at room temperature for 30 min (45 min for CYP2D6), 50 *μ*L of the reconstituted luciferin detection reagent was added to stop the reaction and produce the luminescent signal. The luminescence in all of the samples was measured using a microplate reader as relative light units (RLU) after 20 min incubation to stabilize the luminescent signal. The assay experiments were repeated three independent times.

### 2.3. Statistical Analysis

The amount of light produced is directly proportional to the CYP enzyme activity. The net signals from untreated CYP reactions represent total CYP activity (100%). The modulation of the CYP activity by the test compound was determined by comparing the changes from the average net signal of untreated CYP reactions with the changes observed from the test compound. The IC_50_ values showing more than 50% inhibition were calculated by plotting the percent inhibition of CYPs enzyme activities versus log concentration of the extract or compounds using GraphPad Prism (version 5.01. USA).

## 3. Results

The inhibitory potencies of aRVS extract and the constituents against CYP450 were determined by evaluating the IC_50_ values. The potency of a test compound can be classified by its IC_50_ value according to the following scale: potent if IC_50_ < 20 *μ*g/mL, moderate if IC_50_ = 20–100 *μ*g/mL, or weak if IC_50_ > 100 *μ*g/mL [26]. The aRVS extract was found to exhibit potent inhibitory activity against CYP2C9, CYP2C19, and CYP1A2 (Table 1), with most potent inhibition on CYP2C9 (IC_50_ < 0.001 *μ*g/mL). Among the components, fisetin showed much more inhibitory activity against all the CYPs compared to fustin. Like aRVS extract, fisetin was found to exhibit potent inhibitory activity against CYP2C19, CYP1A2, and CYP2C9 (Table 1), with most potent inhibition on CYP2C19 (IC_50_ < 0.001 *μ*g/mL). However, like aRVS, fisetin showed weak inhibition towards the CYP3A4 and CYP2D6 (Table 1).

## 4. Discussion

The recombinant human CYP enzyme study found that aRVS extract potently inhibits CYP2C9 and so less CYP2C19 and CYP1A2 and weakly inhibits CYP3A4 and CYP2D6. Moreover, the major flavonoid, fisetin, potently inhibits CYP2C19 and so less CYP1A2 and CYP2C9, moderately inhibits CYP2D6, and weakly inhibits CYP3A4. On the other hand, fustin showed negligible inhibition towards the CYP enzymes except CYP2C19. The above result suggests that the tested fisetin is responsible for the inhibition of the CYP isoforms by aRVS extract.

CYP inhibition can lead to higher levels of the cytotoxic drug, causing recognizable, greater toxicity, while inactive prodrugs such as cyclophosphamide or ifosfamide often lead to therapeutic failure because of lower plasma levels of the chemotherapeutic drug [2, 6]. Among the CYP enzymes, CYP3A4 and CYP2D6 are the most important enzymes in the metabolism of anticancer drugs [1, 2]. CYP3A4, which is the most abundant enzyme (~80%) in the intestinal mucosa, should be the most important contributor in drug metabolism [28]. Since most herbal medicines including aRVS extract are administered orally, a high concentration in the gut might cause significant inhibition of CYP3A4. However, our results suggest that aRVS extract and the tested compounds are not likely to inhibit the metabolism of a chemotherapeutic drug whose primary route of elimination is through CYP3A4 or CYP2D6.

CYP2C9, which comprises about 15% of the CYP in the intestinal mucosa, is involved in the metabolism of some chemotherapeutic drugs such as capecitabine, cyclophosphamide, and ifosfamide, drugs with a narrow therapeutic index such as warfarin and phenytoin, and other drugs such as acenocoumarol, tolbutamide, losartan, glipizide, and some nonsteroidal anti-inflammatory drugs [2, 4, 29]. Flavones and flavonols are two major classes of flavonoids, polyphenolic secondary metabolites, which also act as potent inhibitors of CYP2C9 [30].

In our study, fustin (2-(3,4-dihydroxyphenyl)-3,7-dihydroxy-2,3-dihydrochromen-4-one) is a flavanonol, a type of flavonoid. The fisetin (2-(3,4-dihydroxyphenyl)-3,7-dihydroxychromen-4-one) is a flavonol, a structurally distinct chemical substance that belongs to the flavonoid group of polyphenols. It was reported to have multiple pharmaceutical properties such as antiaging, anti-inflammatory, anticancer, and antiviral effects in different lines of culture cells [11, 31]. Several herbs have been reported to show the inhibition of CYP2C19 as well as CYP2C9 [26, 32]. Therefore, inhibition of CYP2C9 and CYP2C19 by aRVS extract is mainly due to the fisetin.

Furthermore, the genetically polymorphic CYP2C9 and CYP2C19 show different drug excretion rates and final serum concentrations in different people. An extensive metabolizer has two copies of wild-type alleles, whereas poor metabolizers have two copies of variant alleles, causing reduced enzymatic activity [33, 34]. The CYP2C9∗2 and CYP2C9∗3, recognized as main CYP2C9 variants in humans, have reduced catalytic activity compared with the wild-type CYP2C9∗1. The CYP2C19∗2 and CYP2C19∗3 variant alleles account for the majority of the poor metabolizer phenotypes. Considering that poor metabolizers with CYP2C9 and CYP2C19 variants are more prominent in Asian population (15–20%) than in Caucasians and African-American populations (3–5%) [35], further inhibition of CYP2C9 and CYP2C19 by herbal medicine, including aRVS extract, likely cause clinically significant herb-drug interactions in the Asian population.

CYP1A2 is able to metabolize some polycyclic aromatic hydrocarbons (PAHs), a procarcinogen, to carcinogenic intermediates, so that higher CYP1A2 activity may influence the risk of lung cancer and breast cancer [36, 37]. CYP1A2 is also involved in the metabolism of antidepressants drugs and antipsychotic drugs [29]. Dietary flavonoids are important contributors for cancer prevention, due to their inhibition of CYP1A2 activity [38]. Therefore, the mechanism of the anticancer effect from aRVS extract in practice might be explained in the view of CYP1A2 inhibition or its genetic polymorphism.

Our in vitro results suggested that aRVS extract potently inhibits CYP2C9, CYP2C19, and CYP1A2, which means aRVS extract in conjunction with chemotherapeutic drugs such as capecitabine, cyclophosphamide, or ifosfamide, or the drugs mentioned above could increase or decrease the plasma level of the drugs, which could result in significant clinical consequences. In contrast, the concurrent administration of aRVS and cisplatin was reported not to alter the antitumor efficacy of cisplatin. In addition, aRVS prevents cisplatin-induced toxicity in vitro and in vivo via an antioxidant activity [39].

Therefore, in vivo investigation of aRVS extract's inhibition of CYP2C9 and CYP2C19 is needed to study drug-herb interaction. The genetic polymorphism of CYP2C9 and CYP2C19 should also be studied, because a better understanding will instruct individuals with ultraextensive or poor metabolism on clinically important herb-drug interactions.

## Figures and Tables

**Table 1:** The IC_50_ values of the aRVS extract, fustin, and fisetin (*μ*g/mL).

	aRVS	Fustin	Fisetin
CYP1A2	10.0	4930.0	0.46
CYP2C9	<0.001	540.2	5.4
CYP2C19	9.68	64.3	<0.001
CYP2D6	503.3	5346.1	46.8
CYP3A4	391.8	463.0	309.0
